# SS-31 treatment ameliorates cardiac mitochondrial morphology and defective mitophagy in a murine model of Barth syndrome

**DOI:** 10.1038/s41598-024-64368-y

**Published:** 2024-06-13

**Authors:** Silvia Russo, Domenico De Rasmo, Roberta Rossi, Anna Signorile, Simona Lobasso

**Affiliations:** 1 Department of Translational Biomedicine and Neuroscience (DiBraiN), University of Bari Aldo Moro, Pl. G. Cesare 11, 70124 Bari, Italy; 2 Institute of Biomembranes, Bioenergetics and Molecular Biotechnologies (IBIOM) , National Research Council (CNR), Bari, Italy; 3 Department of Precision and Regenerative Medicine and Ionian Area (DiMePRe-J), University of Bari Aldo Moro, Bari, Italy

**Keywords:** Metabolic diseases, Mitochondrial proteins, Energy metabolism

## Abstract

Barth syndrome (BTHS) is a lethal rare genetic disorder, which results in cardiac dysfunction, severe skeletal muscle weakness, immune issues and growth delay. Mutations in the *TAFAZZIN* gene, which is responsible for the remodeling of the phospholipid cardiolipin (CL), lead to abnormalities in mitochondrial membrane, including alteration of mature CL acyl composition and the presence of monolysocardiolipin (MLCL). The dramatic increase in the MLCL/CL ratio is the hallmark of patients with BTHS, which is associated with mitochondrial bioenergetics dysfunction and altered membrane ultrastructure. There are currently no specific therapies for BTHS. Here, we showed that cardiac mitochondria isolated from *TAFAZZIN* knockdown (Taz^KD^) mice presented abnormal ultrastructural membrane morphology, accumulation of vacuoles, pro-fission conditions and defective mitophagy. Interestingly, we found that in vivo treatment of Taz^KD^ mice with a CL-targeted small peptide (named SS-31) was able to restore mitochondrial morphology in tafazzin-deficient heart by affecting specific proteins involved in dynamic process and mitophagy. This agrees with our previous data showing an improvement in mitochondrial respiratory efficiency associated with increased supercomplex organization in Taz^KD^ mice under the same pharmacological treatment. Taken together our findings confirm the beneficial effect of SS-31 in the amelioration of tafazzin-deficient dysfunctional mitochondria in a BTHS animal model.

## Introduction

Barth syndrome (BTHS, MIM 302060) is a rare X-linked disease characterized by huge variability of clinical symptoms, primarily including cardiomyopathy, skeletal muscle weakness, delayed growth, organic aciduria, and low level of neutrophils^1–5^. BTHS is caused by loss-of-function mutations in the TAFAZZIN gene, which encodes for an acyltransferase enzyme (tafazzin) involved in the biosynthesis/remodeling of the dimeric phospholipid cardiolipin (CL)^6,7^.

Indeed, defects in tafazzin lead to an alteration of CL composition with a specific increase in the monolysocardiolipin (MLCL)/CL ratio and in the content of immature CL species (containing more saturated fatty acids) together with lower levels of mature CL species (mainly tetralinoleoyl CL in heart, skeletal muscle and liver)^7–9^. As CL represents a key phospholipid for mitochondrial membrane, these abnormalities in CL composition drastically affect mitochondrial structure and function^10–12^.

An outer membrane (OM) and an inner membrane (IM) structurally form the mitochondria. The OM is important for interactions with other cell organelles, including the sarcoplasmic reticulum and the lysosomes. The IM separates the mitochondrial intermembrane space from the matrix compartment and forms mitochondrial cristae, containing the enzymes for mitochondrial respiration and ATP production. The IM contains large amounts of CL and alteration of its biosynthesis has been implicated in impaired mitochondrial energy metabolism associated with increased reactive oxygen species (ROS) production, respiratory chain complexes instability, and defects in mitochondrial biogenesis and dynamics^13–15^.

Mitochondrial dynamics encompass cristae remodeling, fusion and fission of mitochondria, forming a dynamic mitochondrial network that provides an adaptation to cellular metabolic changes, preserves cell integrity, and is involved in selective autophagy (mitophagy)^16–18^.

Mitochondrial fission is mainly regulated by fission protein 1 (FIS1) and cytosolic GTPases dynamin-related protein 1 (DRP1). Several proteins and mechanisms control DRP1 activity and its recruitment to mitochondria in order to regulate fission process^19,20^.

Mitochondrial fusion is mainly mediated by OM-localized GTPases, mitofusins (MFN1 and MFN2), and an IM-localized GTPase, optic atrophy 1 (OPA1). OPA1 protein exists as a long form (L-OPA1) and a short form (S-OPA1) derived from its proteolytic processing by different proteases, such as OMA1 and  YME1L^16^. Recently findings have shown a cooperation between OPA1 and CL in IM fusion process^21^. OPA1 stability is also regulated by prohibitins proteins (PHB1/2); in fact, interaction between PHBs complex and mitochondrial proteases inhibits OPA1 processing favoring mitochondrial fusion and cristae remodeling^22^.

The exact molecular mechanisms by which defects in tafazzin protein and changes in CL composition (and/or enrichment in MLCL content) dramatically affect mitochondrial function in BTHS are not so well understood. Anyway, alterations in mitochondrial dynamics and quality control have been previously reported in BTHS patients. In particular, previous studies revealed that CL alterations were associated with abnormal mitochondrial morphology and impaired mitochondrial function in BTHS patient tissues and animal models^23–26^. On the other hand, a key role of CL in mitochondrial dynamics as well as in removing damaged mitochondria via mitophagy has been elucidated^12,27^.

Thus, understanding the intricate relationship between mitochondrial dynamics and BTHS is considered of great interest for therapeutic strategies. Despite of intensive research efforts in last years, there is still no specific cure for this syndrome to date. Nowadays one of the most promising therapeutic approaches is the CL-targeted drug named SS-31, a water-soluble tetrapeptide that localizes in the mitochondrial IM, resulting in improved membrane organization, cellular respiration, and energy production^28–30^.

A few years ago, SS-31 (also known as elamipretide) was tested in an American clinical trial as a potential first therapy for BTHS patients. The collected data showed that a long exposure to the peptide (48 weeks) resulted in a significant improvement in performance on the 6-Minute Walk Test and BTHS Symptom Assessment Scale, as well as in heart physiology (i.e. increase in cardiac stroke volumes)^31,32^.

Recently, our group has shown that treatment with SS-31 had significant beneficial effects on cardiac mitochondrial dysfunction in a BTHS rodent model (doxycycline-inducible TAFAZZIN knockdown mice, Taz^KD^)^33^. In particular, cardiac mitochondria isolated from Taz^KD^ mice presented an improved mitochondrial respiratory chain efficiency after long-term treatment with SS-31 compared to Taz^KD^ untreated mice, without changes in the phospholipid profile and the typical increased MLCL/CL ratio. Interestingly, this improvement was associated with an increased organization of the mitochondrial respiratory chain into supercomplexes^33^, even if the underlying mechanisms that lead to this functional improvement of the mitochondria remained to be elucidated.

Considering that (1) SS-31 enters the mitochondria where it binds to CL (and MLCL if present) in IM^28,34^, and (2) CL plays a pivotal role in mitochondrial cristae organization^15,35^, in this study we investigated the effects of the CL-targeted peptide on the defective mitophagy and structural organization of tafazzin-deficient cardiac mitochondria.

Here, using the previously reported rodent BTHS model^33^ we found a significant change in mitochondrial structure and demonstrated that SS-31 was also able to restore morphology in the tafazzin-deficient mitochondria by acting indeed on some proteins involved in dynamic process and mitophagy.

## Results

### Treatment with SS-31 mitigates mitochondrial morphology alteration in Taz^KD^ mice

**Figure 1:**
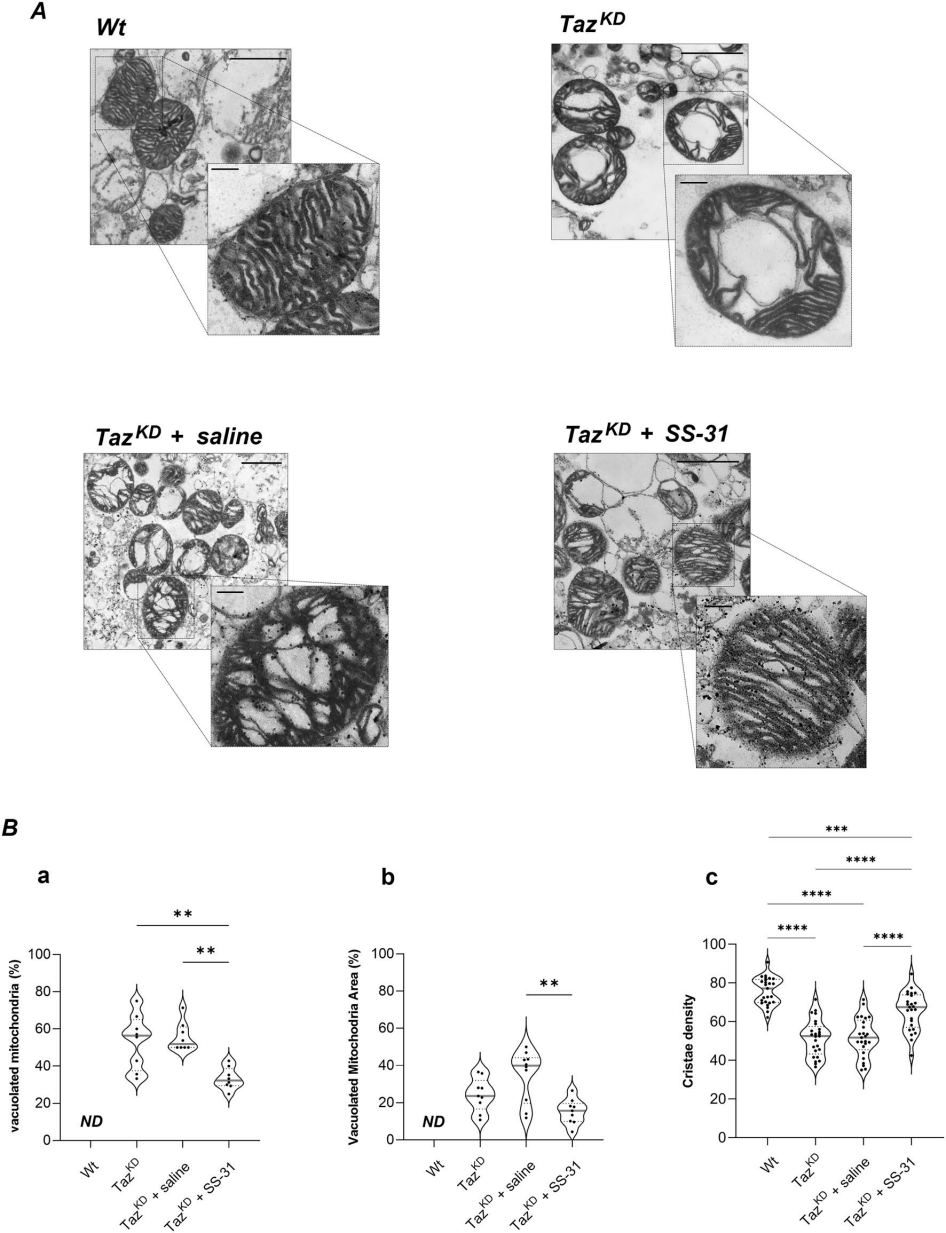
Transmission Electron Microscopy (TEM) analysis of mitochondrial morphology. (**A**) Representative TEM images of ultra-thin sections of cardiac mitochondria isolated from Wt, Taz^KD^, Taz^KD^ + saline and Taz^KD^ + SS-31 mice (14,000X, scale bar = 1 μm). Magnifications (56,000X, scale bar = 0.2 μm) are also shown. (**B**) The violin plots represent the median of values and quartiles of: (**a)** the number of vacuolated mitochondria expressed as percentage of total number of mitochondria, (**b**) the vacuoles area expressed as percentage of total mitochondrial area, and (**c)** the cristae density expressed as percentage of the mitochondrial area occupied by cristae, in the four groups of animals. *p*-value < 0.05 was considered as statistically significant (one-way ANOVA or Kruskal–Walli’s test, **p < 0.01; ***p < 0.001; ****p < 0.0001).

In order to study ultrastructural organization and morphology of mitochondria, we performed transmission electron microscopy (TEM) of cardiac mitochondria isolated from wild-type (Wt) and Taz^KD^ mice (Fig. 1A, upper panels), and from Taz^KD^ mice treated with saline or SS-31 peptide (Fig. 1A, lower panels).

Comparative TEM analysis revealed a severe alteration of mitochondrial morphology in TAZ^KD^ mice. Specifically, tafazzin-deficiency was associated with a massive accumulation of the number of mitochondria, whose area was mainly occupied by putative vacuoles and characterized by a loss in cristae canonical structure and lower density, compared to mitochondria from Wt mice (Fig. 1A upper panels, quantified in Fig. 1B).

In contrast, these morphological alterations were significantly mitigated by long-term treatment of TAZ^KD^ mice with SS-31. In particular, the administration of the peptide elicited a significant reduction in the percentage of vacuolated mitochondria and the area occupied by autophagic vacuoles compared to mitochondria isolated from saline-treated Taz^KD^ mice (Fig. 1A, lower panels, quantified in Fig. 1B(a, b)). Moreover, TEM analysis at higher magnification of Taz^KD^ mitochondrial ultrastructure showed ameliorated structure and density of cristae in Taz^KD^ mitochondria after SS-31 treatment, compared to saline treatment (see Fig. 1A, quantified in Fig. 1B(c)).

These findings suggest that SS-31 treatment mitigates the mitochondrial structural alteration and authophagic vacuolization, leading to normalization of architecture of Taz^KD^ isolated mitochondria.

### Alteration of mitochondrial dynamics in heart of Taz^KD^ mice is ameliorated by SS-31

**Figure 2:**
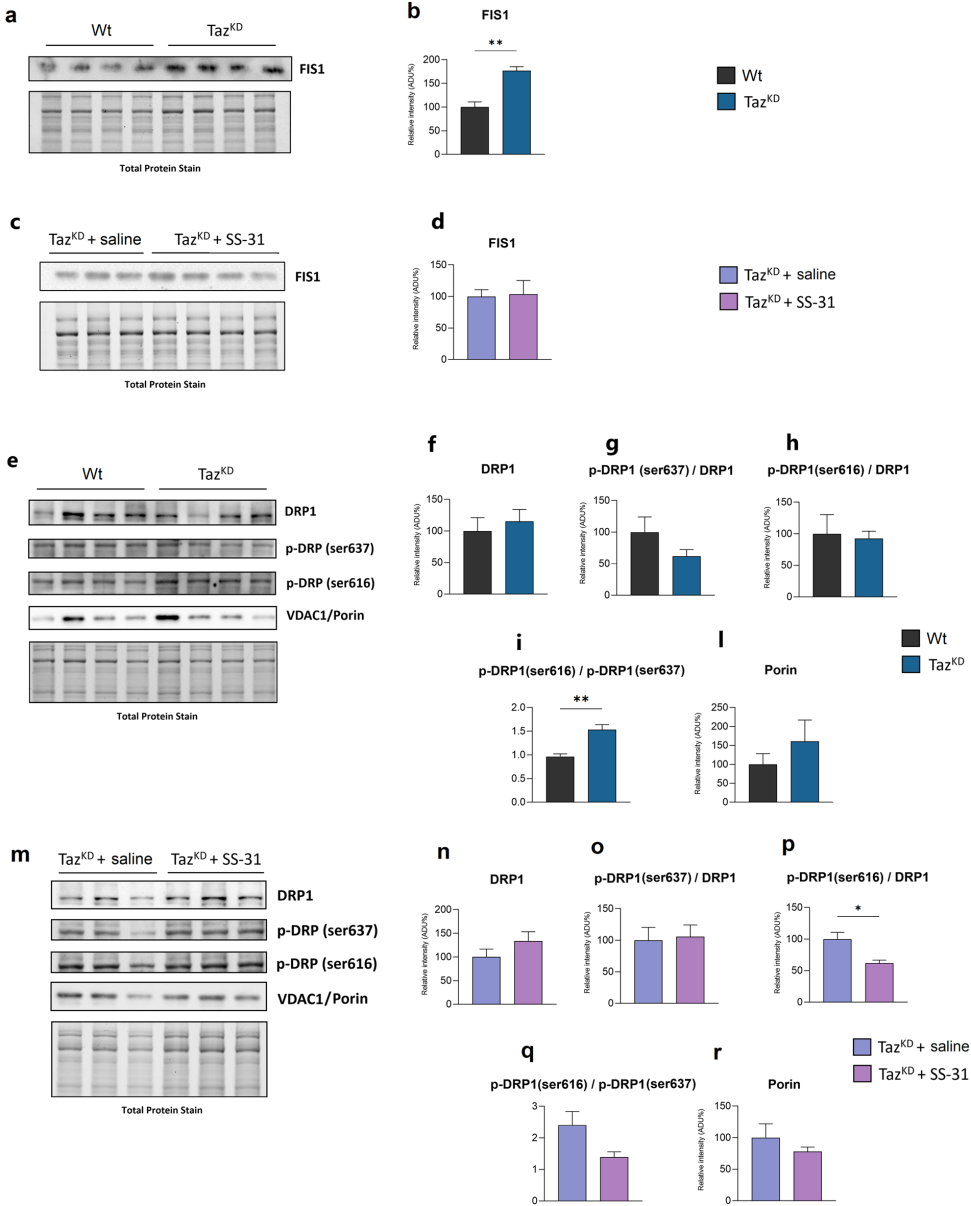
Immunoblotting analysis of proteins involved in mitochondrial fission. Western blot analysis of FIS1, DRP1, p-DRP1(s637), p-DRP1(s616) and porin protein expression in the heart of Wt and Taz^KD^ mice (**a, e**), and Taz^KD^ + saline and Taz^KD^ + SS-31 mice (**c, m**). Isolated cardiac mitochondria (for **a, c**) or total heart homogenates (for **e, m**) were separated by SDS-PAGE, transferred onto PVDF membrane, and then subjected to immunoblotting analysis with specific antibodies, as indicated. The histograms show quantification of the protein expression levels: FIS1 (**b, d)**, DRP1 (**f, n**), p-DRP1(s637)/DRP1 ratio (**g, o**), p-DRP1(s616)/DRP1 ratio (**h, p**), p-DRP1(s616)/p-DRP1(s637) ratio (**i, q**) , porin (**l, r**), in the heart from the four groups of animals, as indicated. Data are presented as means + SEM; n = 3–4 mice per group. Taz^KD^ and Taz^KD^ + SS-31 data are represented as the percentage change from Wt and Taz^KD^ + saline, respectively, set on 100%. *p*-value less than 0.05 was considered as statistically significant (Student’s *t* test, *p < 0.05, **p < 0.01). Original blots are presented in Supplementary Figure S1.

**Figure 3:**
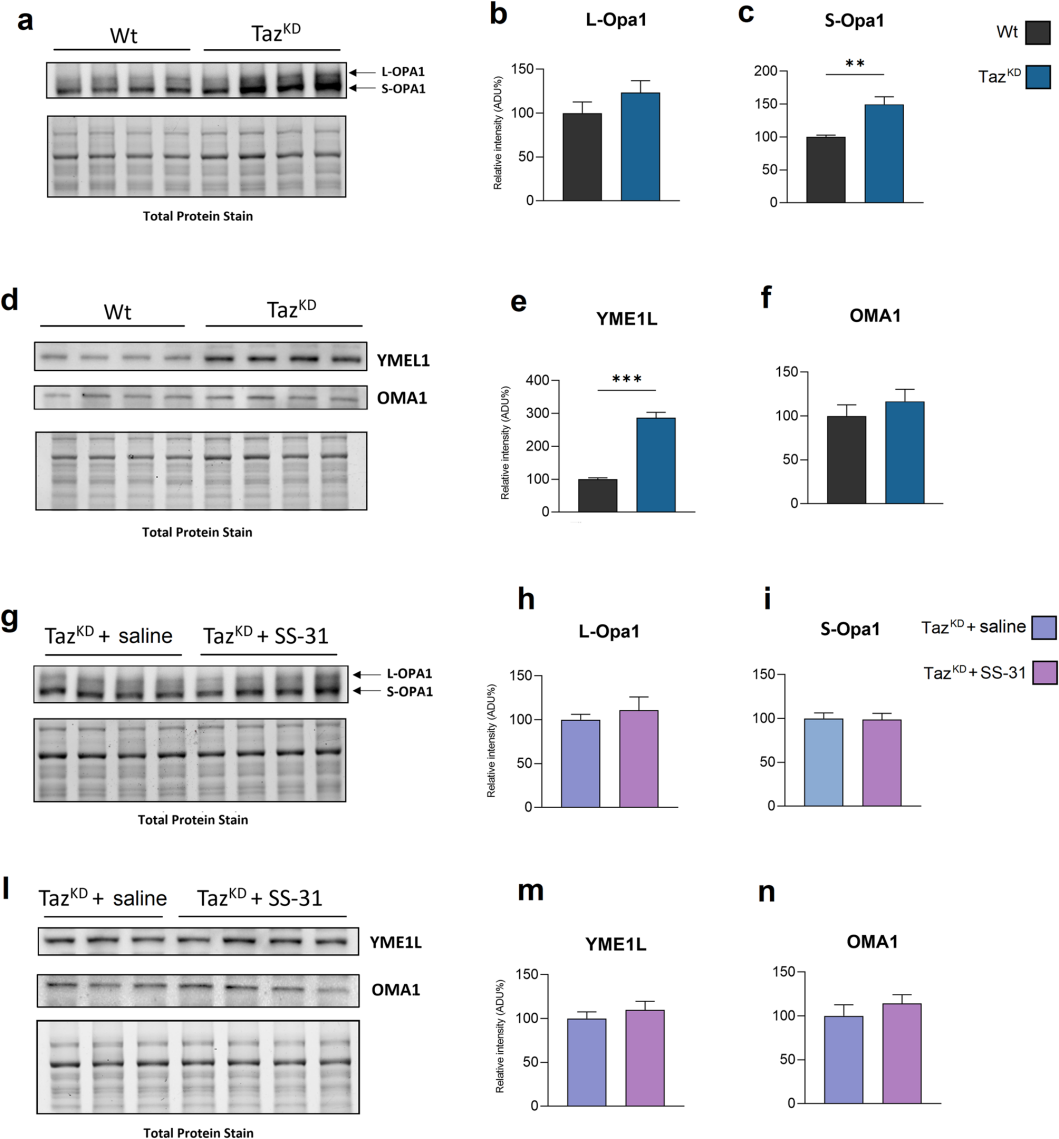
Immunoblotting analysis of proteins involved in mitochondrial fusion. Western blot analysis of L-Opa1, S-Opa1, YME1L, OMA1 protein expression in the heart of Wt and Taz^KD^ (**a, d**), and Taz^KD^ + saline and Taz^KD^ + SS-31 mice (**g, l**). The histograms show quantification of the protein expression levels: L-Opa1 (**b, h**), S-Opa 1 (**c, i**), YME1L (**e, m**) and OMA1 (**f, n**), in cardiac mitochondria from the four groups of animals, as indicated. Data are presented as means + SEM; n = 3–4 mice per group. Taz^KD^ and Taz^KD^ + SS-31 data are represented as the percentage change from Wt and Taz^KD^ + SS-saline, respectively, set on 100%. *p*-value less than 0.05 was considered as statistically significant (Student’s t test, **p < 0.01; ***p < 0.001). Original blots are presented in Supplementary Figure S2.

The above-described changes in mitochondrial ultrastructure and the beneficial effect of SS-31 prompted us to investigate on the molecular aspects involved in these processes. First, we analysed the expression of FIS1, DRP1 and OPA1 proteins involved in mitochondrial dynamics (Figs. 2, 3). The mitochondrial FIS1 protein promotes mitochondrial fission by interacting with DRP1, whose reversible phosphorylation controls its recruitment to mitochondria. Specifically, DRP1 phosphorylation at Ser-616 promotes mitochondrial localization of DRP1 and thus fission^36^, whereas its phosphorylation at Ser-637 inhibits mitochondrial localization reducing fission^37^.

The immunoblot analysis of FIS1 expression showed a higher protein level in isolated Taz^KD^ cardiac mitochondria than in Wt (Fig. 2 A, B), while SS-31 treatment sowed no effect on Taz^KD^ mice with respect to untreated ones (Fig. 2 C, D).

The immunoblot analysis of DRP1 in Taz^KD^ heart homogenates displayed high variability in total DRP1 protein level with no significant differences compared to Wt (Fig. 2 E, F). Since DRP1 activity also depends on its phosphorylation status, specific DRP1 phosphorylations at Ser-616 (DRP1pS616) and Ser-637 (DRP1pS637) have been investigated. The obtained results revealed that neither the DRP1pS637 nor the DRP1pS616 were significantly affected in Taz^KD^ heart homogenates in comparison to control mice (Fig. 2  E, G, H). However, although tafazzin-deficiency only tended to decrease DRP1 phosphorylation at Ser-637 without any alteration in DRP1 phosphorylation at Ser-616, the ratio between DRP1pS616 and DRP1pS637 was statistically higher in Taz^KD^ heart homogenates than in Wt (Fig. 2I) indicating a pro-fission condition in mitochondria. Treatment with SS-31 of Taz^KD^ mice did not alter DRP1 level (Fig. 2 M, N), but significantly decreased the DRP1pS616 level (Fig. 2P), even if the DRP1pS616/DRP1pS637 ratio displayed only a tendency to decrease (Fig. 2 O, P, Q). The immunoblot analysis of VDAC1/porin level performed on heart homogenates showed no significant differences either between Wt and Taz^KD^ mice, or between SS-31-treated and saline-treated Taz^KD^ mice (Fig. 2 E, L, M, R).

As said above, mitochondrial fusion is mainly controlled by OPA1 protein, whose activity depends on the balance between the L and S variants of OPA1^16^. The immunoblots reported in Fig. 3 A–C show that Taz^KD^ mitochondria were characterized by a significant increase of S-OPA1 level, without alteration in L-OPA1 variant compared with Wt. This process could be mainly ascribed to YME1L or OMA1 proteases. Infact, the analysis of YME1L protein expression revealed a significant increase of YME1L protein level in Taz^KD^ mitochondria, while OMA1 expression seemed to remain unchanged (Fig. 3 D–F). SS-31 treatment did not elicit any significant effect on the expression of these two proteases, and on the long or short OPA 1 variants in Taz^KD^ mitochondria (Fig. 3G–N).

### Defective mitophagy in heart of Taz^KD^ mice is improved by SS-31

**Figure 4:**
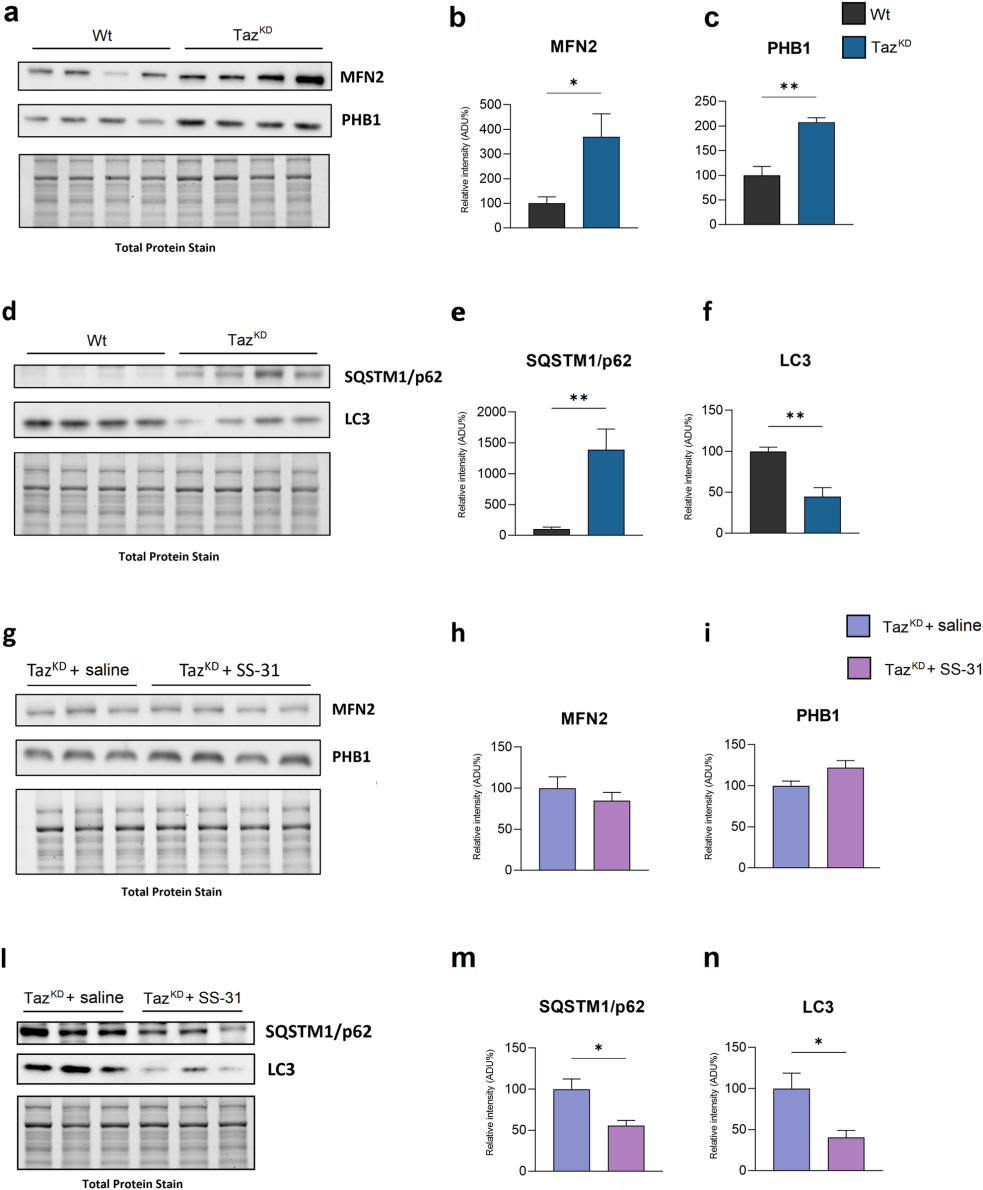
Immunoblotting analysis of proteins involved in mitophagy. Western blot analysis of MFN2, PHB1, SQSTM1/p62, and LC3 protein expression in the heart of Wt and Taz^KD^ (**a, d**), and Taz^KD^ + saline and Taz^KD^ + SS-31 mice (**g, l**). The histograms show quantification of the protein expression levels MFN2 (**b,h**), PHB1 (**c,i**), SQSTM1/p62 (**e, m**) and LC3-II (**f, n**), in cardiac mitochondria from the four groups of animals, as indicated. Data are presented as means + SEM; n = 3–4 mice per group. Taz^KD^ and Taz^KD^ + SS-31 data are represented as the percentage change from Wt and Taz^KD^ + SS-saline, respectively, set on 100% (Student’s *t* test, *p < 0.05; **p < 0.01). Original blots are presented in Supplementary Figure S3.

Considering the previously described accumulation of vacuolated mitochondria (see Fig. 1) and the known role of YME1L protease activity in mitophagy^38^, we decided to investigate some proteins involved in mitophagy and the effect of SS-31 treatment. First, we analyzed the expression of MNF2 and PHB1, which are both multifunctional proteins involved in mitochondrial fusion and mitophagy^22^. Taz^KD^ mice showed an increase in MNF2 and PHB1 protein levels in cardiac mitochondria compared to Wt mice (Fig. 4A–C). Moreover, SQSTM1/p62, which acts as a selective autophagy receptor, showed a significant higher protein level in Taz^KD^ mitochondria than in Wt (Fig. 4 D, E). On the contrary, the expression of autophagosome adapter microtubule-associated protein 1 light chain 3 beta (LC3-II), which is recruited to mitochondria by SQSTM1/p62^39^, was reduced in Taz^KD^ mitochondria compared to Wt (Fig. 4 D, F).

Treatment with SS-31 had no effect on the levels of MNF2 and PHB1 proteins in Taz^KD^ mitochondria (Fig. 4 G–I), but it had an important role on the final stages of mitochondrial recognition for mitophagy. In fact, SS-31-treated Taz^KD^ mice showed significantly lower SQSTM1/p62 protein levels than saline-treated mice, as well as a lower LC3II protein content in isolated mitochondria (Fig. 4 L, M, N).

These findings suggest that treatment of Taz^KD^ mice with SS-31 ameliorates defective mitophagy in heart.

## Discussion

Tafazzin deficiency in BTHS causes several mitochondrial dysfunctions due to abnormalities in CL composition, the key phospholipid for mitochondrial biology. Alterations in mitochondrial morphology, dynamics, respiration and energy production contribute to the overall energy deficiency observed in BTHS patients, primarily in heart and skeletal muscle^40,41^. Moreover, the disruption of mitochondrial dynamics is associated with increased ROS production and altered calcium handling in BTHS^26,41^.

SS-31 is a tetrapeptide belonging to the Szeto-Schiller family that localizes in CL-rich IM, improving mitochondrial function in different organs, including heart, kidney, neurons, and skeletal muscle^29,34,42^. In particular, SS-31 has been found to be involved in the mitigation of oxidative stress and inflammatory processes, in the prevention of cellular apoptosis and in the maintenance of mitochondrial dynamics^43^. Therefore, its potential benefits are of particular interest in the field of diseases associated with mitochondrial dysfunction and in defense mechanisms against oxidative stress-induced metabolic and degenerative diseases^44–46^.

Recently, a beneficial effect of SS-31 on cardiac mitochondrial dysfunction in Taz^KD^ mice has been associated with increased assembly of mitochondrial respiratory chain complexes in supercomplexes^33^.

In general, the improvement of mitochondrial function is often associated with the amelioration of mitochondrial morphology and membrane ultrastructure. So that, in this work we obtained interesting data on mitochondrial morphology and structure in association with molecular aspects of mitochondrial shaping proteins in tafazzin-deficient isolated cardiac mitochondria and, overall, on the effect of long-term administration of SS-31 to Taz^KD^ mice.

In agreement with previous results obtained in heart tissue^24,25^ and isolated mitochondria^47^, TEM analysis of icardiac mitochondria of Taz^KD^ mice suggested altered morphology, mainly characterized by loss in cristae ultrastructure and massive accumulation of large vacuoles. These data, thus, confirm the well-known involvement of phospholipid CL in molecular processes that define mitochondrial morphology and cristae organization^12,13,48^.

Interestingly we showed, for the first time, that long-term SS-31 treatment of Taz^KD^ mice resulted in a reduction of authophagic vacuoles wrapped by mitochondria and a restoration of a control-like mitochondrial morphology. These TEM results in isolated cardiac mitochondria, although still to be confirmed in tissue, are consistent with previous data reporting the positive effect of SS-31 administration on abnormalities of mitochondrial dynamics in left ventricular tissue from both dogs and humans with heart failure^49^.

To evaluate the molecular mechanism at the base of these mitochondrial morphological alterations, we analyzed the expression of some proteins involved in mitochondrial structure and fusion/fission process. Analysis of the fission-mediating proteins FIS1 and DRP1 revealed pro-fission conditions in heart Taz^KD^ mitochondria, as demonstrated by increased level of FIS1. Even if no significant difference has been found in DRP1 protein level, the analysis of DRP1 phosphorylation was also performed. In fact, the recruitment of DRP1 on mitochondrial OM is regulated by its phosphorylation: Ser616 phosphorylation promotes mitochondrial localization of DRP1 and thus fission^36^, whereas the phosphorylation on Ser637 inhibits mitochondrial localization reducing fission^37^. Here we found an increase in the ratio between p-DRP1Ser616 and p-DRP1Ser637 in Taz^KD^ mitochondria compared to Wt supporting pro-fission conditions^50^, in agreement with the higher FIS1 expression previously shown. SS-31 did not affect the increased FIS1 level in Taz^KD^ mice, but by decreasing p-DRP1Ser616 expression as well as the ratio p-DRP1Ser616/Ser637 the treatment proved effective in counteracting pro-fission conditions.

As said before, OPA1 protein is an essential GTPase involved in the modulation of mitochondrial cristae structure^51^, cristae remodeling during apoptosis^52^ and fusion^53^. L-OPA1 form is physically associated with IM by its N-terminal region, while S-OPA1 form is released from L-OPA1 as soluble protein following proteolysis mediated by different proteases in different cellular conditions^54,55^. It is also reported that L-OPA1 and CL cooperate in cristae remodeling and fusion^21^. The relative amounts of L and S forms of OPA1 influence membrane fusion and fission events and an increase in S-OPA1 level leads to an attenuation of mitochondrial fusion^56^.

Our data showed an increased level of S-OPA1 in cardiac mitochondria of Taz^KD^ mice confirming pro-fission conditions. This is also consistent with the observed increased level of YME1L, one of the proteases acting on OPA1 processing^54^. SS-31 treatment had no significant effect on the increased levels of both S-OPA1 and YME1L.

The pro-fission conditions in Taz^KD^ cardiac mitochondria here found and the known role of CL in recognizing damaged mitochondria to eliminate them^57,58^, prompted us to investigate several proteins involved in mitophagy. Indeed, the externalization of CL from the mitochondrial IM to the OM surface represents an important signal for the autophagosome biogenesis, which directs damaged mitochondria to mitophagy^27,59^. Furthermore, it has been demonstrated that CL remodeling by tafazzin is selectively required for the initiation of mitophagy in mouse embryonic fibroblasts and HeLa cells^26,60^. Recently Zhang et al*.* reported that heart and skeletal muscle of Taz^KD^ mice showed impaired mitophagy and rapamycin mitigated mitochondrial dysfunction by blocking MTORC1 signaling^61^.

Regarding mitophagy, several proteins participate to this complex process such as SQSTM1/p62, LC3, PHB2 and MFN2^62–64^. During autophagy, LC3-II associates with the newly formed autophagosome membrane. Damaged mitochondria recruit parkin protein at the mitochondrial OM, where it ubiquitinates SQSTM1/p62 and promotes its binding to LC3-II, thus allowing the recruitment of damaged mitochondria to the phagophore^57^. MFN2 can also act as a mitochondrial OM receptor for parkin, playing a role in promoting mitophagy^65,66^. It is generally accepted that the recognition of damaged mitochondria by LC3-II depends on mitochondrial OM receptor proteins, particularly SQSTM1/p62^67^. In addition, LC3-II can also interact with a protein of IM. Indeed PHB2 has been found to be available for interaction with LC3-II by its interacting domain^22,68^, upon the rupture of the mitochondrial OM through a proteasome-dependent mechanism.

In our experimental model, cardiac mitochondria isolated from Taz^KD^ mice showed increased levels of both mitophagy receptor proteins MFN2 and PHB2, associated with a reduction in LC3-II expression and increased levels of SQSTM1/p62. The amount of LC3-II is closely correlated with the number of autophagosomes, thus a decrease in LC3-II expression suggests a defect in the mitophagy process^69^. In addition, considering that autophagic activation generally leads to SQSTM1/p62 reduction^70^ and that SQSTM1/p62 accumulation is considered a marker of autophagy flux inhibition^67^, the increase in SQSTM1/p62 expression we found confirms a block of mitophagy in Taz^KD^ mitochondria. Interestingly, the SS-31 treatment of Taz^KD^ mice resulted in a significantly decreased level of SQSTM1/ p62, thus indicating a tentative of mitophagy restoration.

Mitochondrial fission is essential for mitophagy and both together coordinately control mitochondrial quality. Even if we found pro-fission conditions in cardiac mitochondria of Taz^KD^ mice, the higher expression of pro-fission proteins is not accompanied by an increase in mitophagy, but rather a block of mitophagy is present, as shown by the accumulation of vacuolated mitochondria, higher level of SQSTM1/p62 and lower expression of LC3-II.

Here, we report for the first time that SS-31 treatment of tafazzin-deficient mice restored morphology of isolated mitochondria acting on some proteins involved in dynamics process. In conclusion, the SS-31 peptide is able to counteract the mitophagy block in tafazzin-deficient heart restoring the mitochondrial functions towards a potential amelioration of cardiomyopathy in BTHS.

Taken together and in agreement with previous data, showing the improvement of mitochondrial respiratory capacity and stabilization of supercomplex organization in Taz^KD^ mice under the same pharmacological treatment^33^, our findings confirm the beneficial effect of SS-31 for improving dysfunctional mitochondria in a BTHS animal model, suggesting the peptide as an effective therapy.

## Methods

### BTHS animal model and SS‑31 treatment

Taz^KD^ mice were obtained by mating transgenic male mice [B6.Cg-Gt(ROSA)26Sor < tm37(H1/tetO-RNAi:Taz)Arte > /ZkhuJ; The Jackson Laboratory, Bar Harbor, ME] for a doxycycline (dox) -inducible TAFAZZIN specific short hairpin RNA (shRNA) with female C57BL/6 J mice (The Jackson Laboratory, Bar Harbor, ME), as previously described^24,25,33^. TAFAZZIN knockdown was induced in utero and maintained postnatally by TD.01306 chow (Envigo, IN, USA) supplemented with 625 mg/kg dox, as previously reported^33^. Only male mice were used for our study. In general, tafazzin expression was found to be approximately 15–20% in cardiac muscle of Taz^KD^ mice compared to dox-fed Wt mice^33^.

SS-31 (New England Peptide Inc, USA) or normal saline as a vehicle was administered to 4-month-old male Taz^KD^ mice with a subcutaneous dose of 3 mg/kg/day for 10 weeks, as previously reported^33^. Taz^KD^ and Wt littermates were sacrificed at 4 months of age, whereas mice that received the SS-31 treatment (or saline) were 10 weeks older (approximately 6.5 months of age).

The animals were kept under a 12-h dark-to-light cycle, constant room temperature and humidity, with food and water ad libitum.

### Heart homogenates preparation and mitochondria isolation

Mice hearts were excised and placed in ice-cold 0.9% NaCl solution. The blood was removed by washing and the connective tissue was cut off. Then the tissues were minced in 2 mL of ice-cold mitochondria isolation buffer (MIB), which was described in our previous study^33^. The minced hearts were gently homogenized using a Dounce glass-Teflon homogenizer. After centrifugation at 800×*g* for 10 min at 4 °C, aliquots of the supernatant were collected and stored at − 80 °C. When indicated, heart homogenates were used for immunoblotting analysis, as described below.

Mitochondria were sedimented by centrifugation of the remaining supernatant at 10,000×*g* for 10 min at 4 °C. The resulting pellet was washed once with MIB and then resuspended in a small volume. The total protein concentration was determined by the Bradford protein assay.

### Transmission electron microscopy (TEM) analysis

Cardiac isolated mitochondria were sedimented by centrifugation at 10,000×*g* for 10 min at 4 °C, the supernatant was removed, and the resulting pellet was fixed in 2.5% glutaraldehyde 0.1 M phosphate buffer (TBS) for 1 h at 4 °C. After washing twice with TBS, samples were post-fixed with 1% osmium tetroxide/TBS for 1 h at 4 °C, dehydrated in a growing series of acetone, and then embedded in Epoxy Resin-Araldite(M) CY212 (TAAB, Aldermaston, UK). Ultra-thin sections were mounted on Formvar-coated nickel grids and stained with uranyl acetate and lead citrate. Sections were examined using a Morgagni 268 transmission electron microscope (FEI Company, Milan, Italy) and images were acquired at 14,000× and 56,000×. Vacuolated mitochondria, expressed as a percentage of total mitochondria, were determined by examining eight fields for each sample (image taken at 14,000×). The total area of mitochondria and the area of their vacuoles were calculated using the “Closed Polygon” function of iTEM-SIS (Soft Imaging System-Olympus). For each sample 10 mitochondria were analysed. For the calculation of mitochondria cristae density, 25 mitochondria were analysed for each sample (image taken at 56,000×). Quantitative analysis was performed with ImageJ software using the thresholding algorithm, based on the separation of high-intensity pixels from low-intensity pixels to distinguish regions of interest (ROI) from background. Cristae density was expressed as the percentage of mitochondrial area occupied by cristae.

### SDS-PAGE and immunoblotting

Proteins (20 μg) from heart homogenates or cardiac isolated mitochondria were solubilized in Laemmli sample buffer (Bio-Rad) and heated at 37 °C for 10 min. Then, samples were run in 7.5 or 10% SDS polyacrylamide gels (BioRad TGX FastCast Acrylamide Kit, #1,610,171, #1,610,173), and transferred to a PVDF membrane by Trans-Blot Turbo Transfer System (Bio-Rad).

The PVDF membranes were blocked in 5% non-fat milk prepared in TBST buffer (0.5 M NaCl, 20 mM Tris, 0.05% Tween 20, pH 7.5) for 1 h at room temperature and then incubated overnight with primary antibodies in 5% non-fat milk TBST at 4 °C, with constant shaking. The blots were then washed three times with TBST for 10 min and incubated with HRP-conjugated secondary antibodies in TBST for 1 h at 4 °C. All antibodies used in this study are listed in the Supplementary Table S1. Reactive proteins were revealed using an enhanced chemiluminescent detection system (Clarity Western ECL Substrate, Bio-Rad) and visualized on a Chemidoc Touch imaging system (Bio-Rad). Densitometric analysis was performed using Image Lab (Bio-Rad), and the relative expression of proteins was normalized with Stain free imaging technology.

### Statistical analysis

Statistical analysis was performed using GraphPad Prism (GraphPad, San Diego, CA, USA). TEM data are presented as violin plots with median and quartiles. First, the Shapiro–Wilk test was used to assess the data distribution, then analysis of variance (one-way ANOVA) followed by Tukey’s post-test was performed for parametric data, while Kruskal–Walli’s test followed by Dunn’s multiple comparison test was performed for non-parametric data.

Immunoblot data are reported as histograms showing the mean value + standard error mean (SEM). Significant differences between data sets were determined using Student’s *t*-test. *p*-value < 0.05 was considered statistically significant.

### Ethical approval

The study is approved by the Ethics Committee of the University of Bari Aldo Moro (OPBA) and by Italian Ministry of Health (Approval Number 162/2020-PR on 5 March 2020) in accordance with internationally accepted guidelines for animal care. The study was also carried out in compliance with the ARRIVE guidelines.

## Data availability 

The datasets generated for this study will be made available on request to the corresponding author.

### Supplementary Information

Supplementary Information.
