# Supplementary material for: SS-31 treatment ameliorates cardiac mitochondrial morphology and defective mitophagy in a murine model of Barth syndrome
Source: Sci Rep. 2024 Jun 13;14:13655. doi: 10.1038/s41598-024-64368-y (PMC11176169; doi:10.1038/s41598-024-64368-y)
Supplement: Supplementary file 1 — Supplementary Information. [file 41598_2024_64368_MOESM1_ESM.pdf]

SUPPLEMENTARY MATERIAL

SS-31 TREATMENT AMELIORATES CARDIAC MITOCHONDRIAL MORPHOLOGY AND DEFECTIVE MITOPHAGY IN A MURINE MODEL OF BARTH SYNDROME

Silvia Russo[1#], Domenico De Rasmo[2#], Roberta Rossi[3], Anna Signorile[1] & Simona Lobasso[1]**

[1]Department of Translational Biomedicine and Neuroscience (DiBraiN), University of Bari Aldo Moro, Bari, Italy; [2]CNR-Institute of Biomembranes, Bioenergetics and Molecular Biotechnologies, Bari, Italy; [3]Department of Precision and Regenerative Medicine and Ionian Area (DiMePReJ), University of Bari Aldo Moro, Bari, Italy

Legend Supplementary Figures 1-3.
Stain free images of full-length gels are shown on the right of each blot. The images show the total proteins used for normalization of each lane. Precision Plus Protein™ All Blue Prestained Protein Standards #1610373 in the range of 10–250 kDa were used to identify protein molecular weights.
Each PVDF membrane was cut into sections with a razor blade, before blotting. Each section was incubated with a different primary antibody, as several of the selected primary antibodies belong to the same species. All blots were exposed separately with a BioRad ChemiDoc Touch Imaging (*Bio-Rad*) to avoid signal saturation. The optimal exposure image selected for each protein is shown. Cropped areas showed in the figures of the manuscript are marked by red lines.

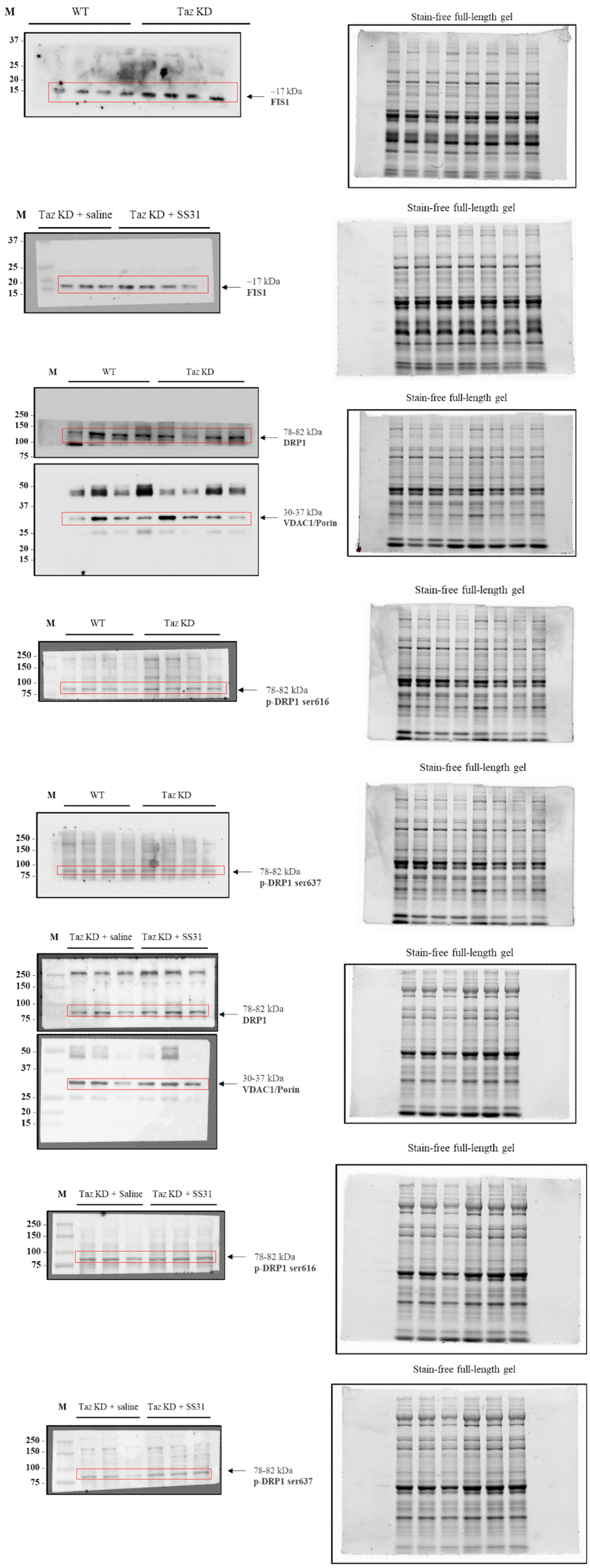
M
WT
Taz KD
37
25
20
15
~17 kDa
FIS1
Stain-free full-length gel
M
Taz KD + saline
Taz KD + SS31
~17 kDa
FIS1
250
150
100
75
78-82 kDa
DRP1
50
30-37 kDa
VDAC1/Porin
78-82 kDa
p-DRP1 ser616
78-82 kDa
p-DRP1 ser637
Taz KD + Saline

Figure S1

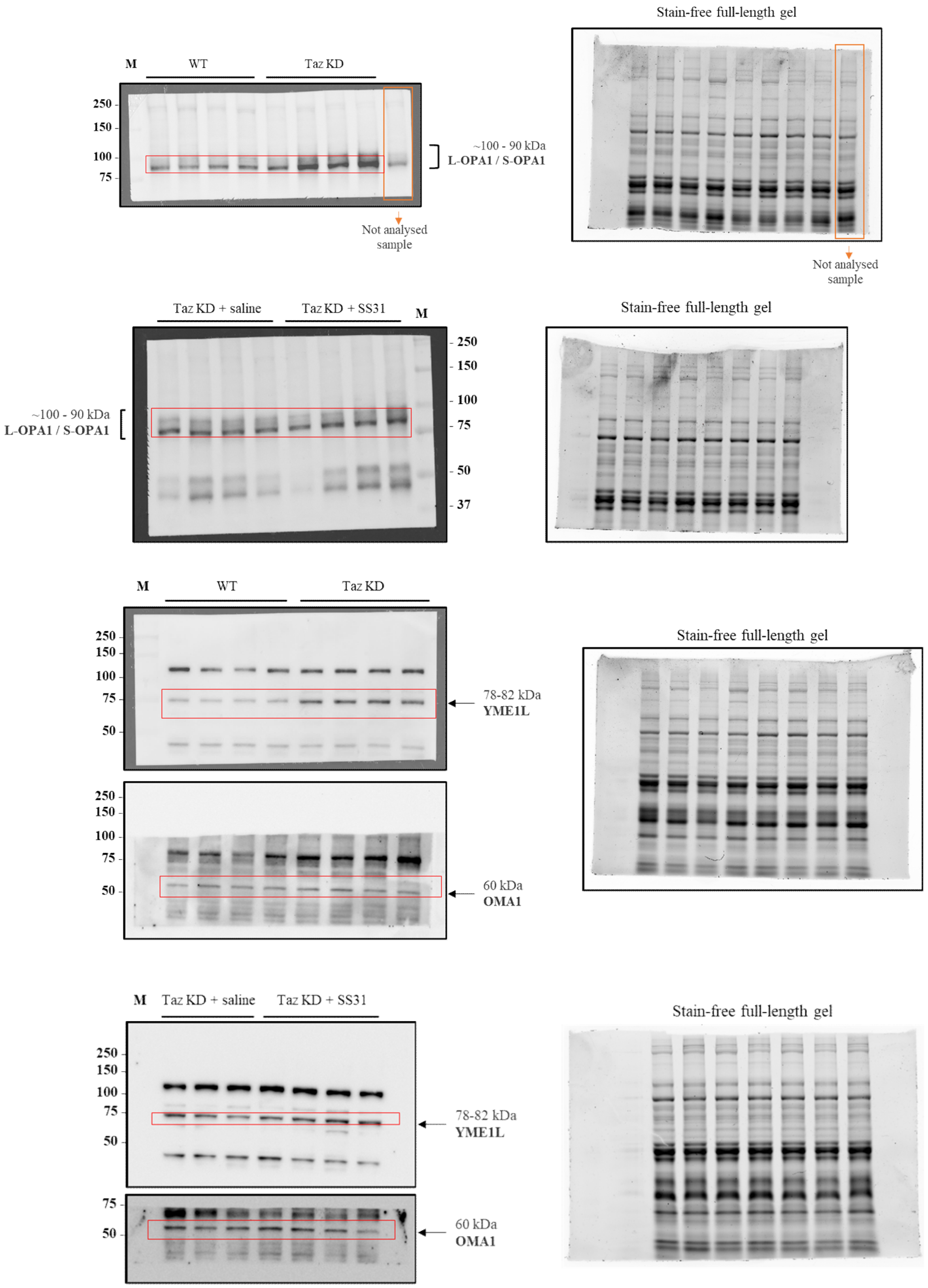

M
WT
Taz KD
250
150
100
75
~100 - 90 kDa
L-OPA1 / S-OPA1
Not analysed sample
Stain-free full-length gel
Not analysed sample
Taz KD + saline
Taz KD + SS31
M
250
150
100
75
50
37
~100 - 90 kDa
L-OPA1 / S-OPA1
Stain-free full-length gel
M
WT
Taz KD
250
150
100
75
50
78-82 kDa
YME1L
250
150
100
75
50
60 kDa
OMA1
Stain-free full-length gel
M
Taz KD + saline
Taz KD + SS31
250
150
100
75
50
78-82 kDa
YME1L
75
50
60 kDa
OMA1
Stain-free full-length gel

Figure S2

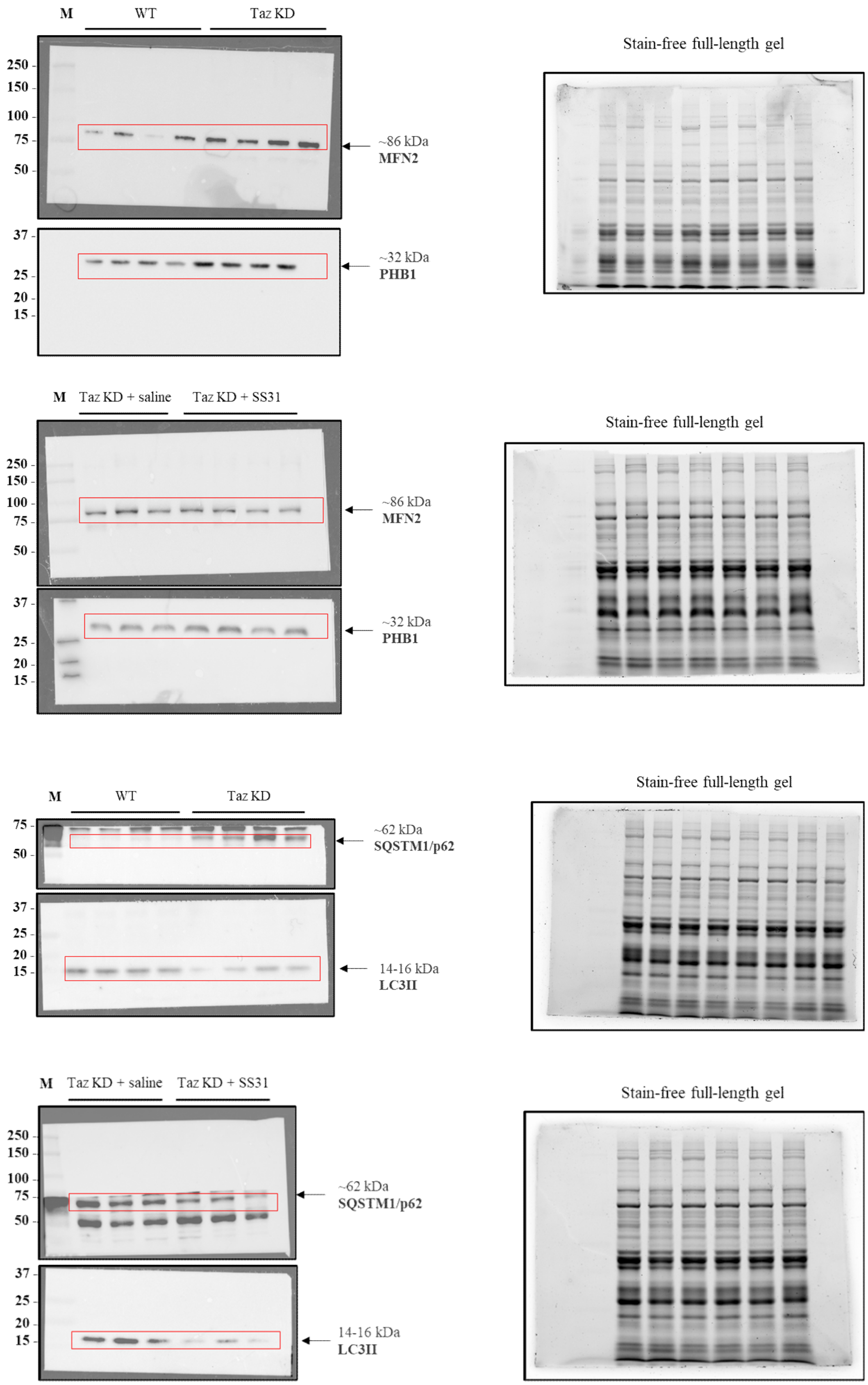

M
WT
Taz KD
250
150
100
75
50
~86 kDa
MFN2
37
25
20
15
~32 kDa
PHB1
Stain-free full-length gel
M
Taz KD + saline
Taz KD + SS31
250
150
100
75
50
~86 kDa
MFN2
37
25
20
15
~32 kDa
PHB1
Stain-free full-length gel
M
WT
Taz KD
75
50
~62 kDa
SQSTM1/p62
37
25
20
15
14-16 kDa
LC3II
Stain-free full-length gel
M
Taz KD + saline
Taz KD + SS31
250
150
100
75
50
~62 kDa
SQSTM1/p62
37
25
20
15
14-16 kDa
LC3II
Stain-free full-length gel

Figure S3

Supplementary Table 1:
List of antibodies used for immunoblotting analysis.

Antibody	Host animal	Dilution	Distributor, Cat. Num.
Anti-FIS1	Rabbit, polyclonal	1:1,000	Merck Millipore, ABC67
Anti-DRP1	Rabbit, polyclonal	1:200	Santa Cruz Biotechnology, Inc, sc-32898
p-DRP1 ser(616)	Rabbit, polyclonal	1:500	Cell Signaling Technology, Inc, 3455
p-DRP1 ser(637)	Rabbit, polyclonal	1:500	Cell Signaling Technology, Inc, 4867
VDAC1/Porin	Mouse, monoclonal	1:1,000	Molecular Probes, A31855
OPA1	Mouse, monoclonal	1:2,000	Thermo Fisher Scientific, Inc, MA5-16149
YME1L	Rabbit, polyclonal	1:2,000	Abcam, ab170123
OMA1	Rabbit, polyclonal	1:500	Abcam, ab154949
MFN2	Rabbit, monoclonal	1:2,000	Cell Signaling Technology, Inc, 11925
PHB1	Rabbit, polyclonal	1:1,000	Cell Signaling Technology, Inc, 2426
SQSTM1/p62	Rabbit, polyclonal	1:1,000	MBL International, PM045
LC3	Rabbit, polyclonal	1:1,000	Cell Signaling Technology, Inc, 2775
